# A Wearable IMU System for Flexible Teleoperation of a Collaborative Industrial Robot

**DOI:** 10.3390/s21175871

**Published:** 2021-08-31

**Authors:** Gašper Škulj, Rok Vrabič, Primož Podržaj

**Affiliations:** Faculty of Mechanical Engineering, University of Ljubljana, Aškerčeva 6, 1000 Ljubljana, Slovenia; gasper.skulj@fs.uni-lj.si (G.Š.); primoz.podrzaj@fs.uni-lj.si (P.P.)

**Keywords:** IMU, wearable system, teleoperation, industrial robot

## Abstract

Increasing the accessibility of collaborative robotics requires interfaces that support intuitive teleoperation. One possibility for an intuitive interface is offered by wearable systems that measure the operator’s movement and use the information for robot control. Such wearable systems should preserve the operator’s movement capabilities and, thus, their ability to flexibly operate in the workspace. This paper presents a novel wireless wearable system that uses only inertial measurement units (IMUs) to determine the orientation of the operator’s upper body parts. An algorithm was developed to transform the measured orientations to movement commands for an industrial collaborative robot. The algorithm includes a calibration procedure, which aligns the coordinate systems of all IMUs, the operator, and the robot, and the transformation of the operator’s relative hand motions to the movement of the robot’s end effector, which takes into account the operator’s orientation relative to the robot. The developed system is demonstrated with an example of an industrial application in which a workpiece needs to be inserted into a fixture. The robot’s motion is compared between the developed system and a standard robot controller. The results confirm that the developed system is intuitive, allows for flexible control, and is robust enough for use in industrial collaborative robotic applications.

## 1. Introduction

The importance and impact of automation and robotics are increasing. Automating tasks using machines increases productivity and improves the comfort of living. The underlying driver is the idea that automating repetitive tasks allows humans to focus on unique activities that require their cognitive abilities. This, however, does not mean that humans and machines should operate separately. On the contrary, future trends envision close human–robot interaction and collaboration. In turn, this requires improved human–robot interfaces that will increase the ease of interaction and allow for greater flexibility. Accurate and timely transformation of human intentions into machine actions is the key factor for improving future automation flexibility.

In general, the process of automating tasks is case-specific and can be performed using various machines. However, six-axis articulated robotic arms—industrial robots—are notable for their flexibility and widespread use. Their design allows them to perform a wide variety of tasks involving precise, repetitive movements. Although industrial robots resemble human arms, their operational characteristics are very different. Industrial robots perform repeated movements without inducing fatigue and can move and hold heavier objects with greater precision. Humans, on the other hand, have the ability to quickly adapt their arm movements to new situations. Therefore, industrial robots can be regarded as highly reliable, whereas humans are highly adaptable.

The reliable motion of industrial robots is achieved through programs created by human programmers. The programs determine the path of the robot, which can depend on external inputs. With the use of additional sensors and algorithms, the robot can be made adaptable, but only limited and case-specific adaptability is currently achievable. Consequently, most industrial robots are only trusted to safely operate in enclosed cells without the presence of a human in their workspace. They are programmed by specialists and tested with limited speeds while the programmer holds a dedicated safety button, the dead man’s switch. If a problem occurs, the robot can be stopped immediately without negative consequences. Safety concerns are the main reason why classical industrial robots are not used alongside human workers.

In contrast to the separation of human and robot workspaces through restricted access and passive and active safety systems, collaborative robots allow humans and robots to share the workspace and the work [1]. Collaborative robots use various mechanisms, such as mechanical compliance, sensorized skin and proprioceptive sensors, to minimize injury in potential collisions with humans, as well as motion capture systems and computer vision to actively avoid collisions [2]. An area where collaborative robots have been especially successfully employed in industry is collaborative assembly, where improvements have been achieved in productivity and quality by collaborative robots and humans working together [3].

Although humans can work alongside collaborative robots, the majority of users are not trained in robot programming and, therefore, the robot will be limited to repetitive motions and simple human–robot interfaces, such as buttons. When the task requires the robot to perform motions that cannot always be pre-programmed in advance, human input can be used to guide the robot. There is a need for intuitive human–robot interfaces that can be employed by a wide range of people, including laymen [4]. This problem is similar to personal computer interfaces, where the users are mainly non-programmers but can still effectively use computers through intuitive interfaces (e.g., graphical user interface, computer mouse, touchpad/screen).

Different possibilities exist, each with distinct advantages and disadvantages, for how to convey the required actions to the robot without programming the specific motion path. There are direct and indirect approaches to changing the position of the robot. When programming a robot indirectly, the interface between the human and the robot needs to interpret the human input and autonomously generate the robot’s motion. An example of an indirect method is gesture control, where the human communicates their desires using gestures, and the robot then autonomously performs appropriate movements based on its programming [5]. Gesture control often uses RGBD cameras for human pose estimation and is often supplemented by voice commands [6,7]. In industrial applications, safety and trust are emphasized as key factors when employing gesture control [8,9]. Direct control, on the other hand, is usually performed using a simple interface such as the robot’s teach pendant, a keyboard, or a joystick. A specific method of direct control is teleoperation, in which the control is performed at a distance.

A distinct option for teleoperation is to use the pose of the operator’s body as the control input [10]. This allows the operator to convey the desired motion to the robot intuitively and in real time, without the need to use the control interface. Several authors address the issues that arise in real-time robot teleoperation, such as delays and tremor, using variable gain [11,12], predictive [13], and fuzzy [14,15] controllers.

In order to generate robot command inputs, the position of the operator’s body has to be captured with appropriate sensors. This can be performed using either a contactless vision-based system or a wearable joint rotation measurement system [16]. The measurement of joint rotation using a wearable system can be achieved using any sensor that can measure changes in position or orientation (e.g., a potentiometer or an encoder), including inertial measurement units (IMUs).

A 6-degree-of-freedom (DOF) IMU consists of an accelerometer and a gyroscope, while a 9-DOF IMU additionally contains a magnetometer. The magnetometer is usually the slowest and the least precise and reliable of the three. Data from the IMUs are usually fused to determine their orientation in space. This is commonly performed using Kalman filters and is employed in arm [17] and hand [18] tracking for human–robot interfaces. The advantageous properties of an IMU-based positional system are its low cost, low power consumption, fast refresh rate, small size, and light-source and line-of-sight independence. IMU suits were proposed as a solution to the problem of unobstructed motion capture as early as 2004 in the context of humanoid robot control [19], and motion capture of specific body parts for specific applications has improved significantly since then. IMU systems for tracking head [20], leg [21], hand [22], and wrist [23] motion have been used in various industrial robotic applications as well as for rehabilitation tasks [24,25].

This paper presents an IMU-based flexible teleoperation system for controlling collaborative robots. The system is composed of several IMUs placed on key points of a human operator’s torso and arms, providing real-time orientation information to a central computer. The computer interprets the information from the IMUs and transforms it into commands for an industrial collaborative robot. While IMU-based systems have been developed for capturing the motion of human limbs [26,27], this paper specifically focuses on capturing torso and arm motion in industrial settings without interfering with the operator’s work, for example, by using sensor gloves. The system presented in [28] enables upper body motion tracking for teleoperation but uses a potentiometer to capture the elbow angle and a glove-like mount for a wrist IMU. Similarly, the system presented in [29] collects upper limb motion features for collaborative robotic applications using markers for system validation but is based on gesture control [6]. Several papers [30,31] survey upper body motion tracking, but they do not address issues related to the control of industrial robots.

The main contributions of this work are, therefore: (1) an IMU-only system for detecting torso and arm motion in industrial settings, without interfering with the work of the operator, that is, without using gloves, special markers, or constricting wearables; (2) a method of transforming the operator’s motion into commands for the robot; and (3) a way to address the practical issues of real-time communication with industrial robot controllers for teleoperation [32,33]. An illustrative case study demonstrates the benefits of the developed system, including its suitability for industrial settings and flexible use for tasks, where the user teleoperates a collaborative robot intermittently to perform support tasks while performing the main task.

## 2. Materials and Methods

The developed system for robot teleoperation connects a human operator with a collaborative robot and enables the use of the robot as a hand-controlled tool. The developed system pursues one main goal:

- The human operator should be able to flexibly manipulate the position and orientation of an object using a collaborative robot by moving their upper body.

The requirement for flexible teleoperation leads to the following specific sub-goals:

- The human operator can freely move around the robot during teleoperation.
- The human operator does not have to think about the transformation of their body movement to the robot’s movement.
- The human operator can easily switch between robot teleoperation and tasks that require the use of their hands.
- The teleoperation system can easily be removed from the workspace.

The listed goals greatly constrain the design space of the robotic teleoperation system. Firstly, because the robot is used to manipulate an object in a structured industrial environment, the human operator is primarily concerned with the position of the robot’s end effector in Cartesian space. If the robot arm were fully anthropomorphic, the change in the operator’s joint positions would be easily transformed to the robot’s joint positions, and the position of the hand/end effector in Cartesian space would be preserved. However, this is not the case, because the industrial robotic arm differs from a human arm in its joint types, link lengths, and range of motion. Therefore, the robot should be controlled in Cartesian space instead of in joint space. The positions of the robot joints are not directly controlled by the operator but are calculated by the robot’s controller with the use of inverse kinematics.

For the operator to freely move around during teleoperation, the wearable system should be battery powered and communicate wirelessly. Battery-powered operation somewhat limits the computational capabilities of the wearable system. More demanding computations should preferably be performed by a system that is not limited by power consumption and size. More importantly, the free movement of the operator changes their relative position with respect to the robot and, thus, their perception of the coordinate system orientation. Therefore, the wearable system should capture the change in the operator’s relative position in order to support intuitive control in Cartesian space.

The ability of the operator to easily and quickly switch from teleoperation to other tasks implies that the wearable system should have a robust process to enable and disable the teleoperation, preferably by using a hand-held button. However, the wearable system should not be attached to the operator’s hands or put strain on them (in contrast to, for example, a typical teach pendant). During teleoperation, the operator should only use their hands to reliably control whether the robot moves or remains still. Because human hands allow for precise control and are very expressive, they are often overused, which can lead to fatigue or injury (e.g., carpal tunnel syndrome).

Finally, the wearable system and its integration with the collaborative robot should be non-invasive and non-destructive so that it is easily removable from the workspace. The wearable system should be worn on top of clothing without the need for precise positioning on the body. The robot should be used without hardware modifications and controlled using default communication channels provided by the manufacturer. These restrictions can facilitate the acceptance and adoption of the developed system for practical industrial applications.

### 2.1. Control System Overview

**Figure 1:**
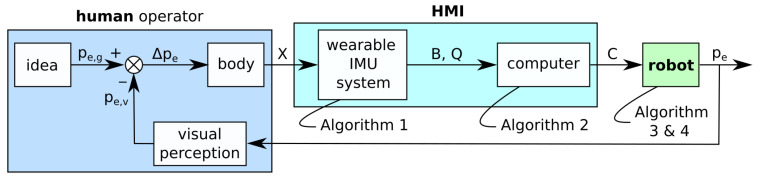
The control system.

The developed system is a position control system with a feedback loop, as shown in Figure 1. The controlled position $p_{e}$ is the position of a useful reference point on the robot’s end effector (e.g., a point between the contact points of the gripper). A well-defined end-effector reference point is important because it has to be easily recognizable by the human operator.

The operator starts with a specific idea of how the end effector should be positioned or what its goal position $p_{e,g}$ should be in the immediate future, defined by t+dt, where dt is loosely defined by human motion perception abilities and ranges between 0.01 s and 0.1 s. The disparity $\Delta p_{e}$ between the goal position $p_{e,g}$ and visually perceived end-effector position $p_{e,v}$ is used by the operator to move into a new position *X*. The movement should ideally be made instinctively.

The pose of the operator’s body *X*, which is used for robot control, is measured by the wearable IMU system. The system measures the orientation *Q* of the operator’s torso, upper arms, and forearms in the form of quaternions. The system also monitors states of buttons *B* that can be pressed with a finger. The wearable system measurements are sent via wireless Local Area Network (LAN) to a computer that interprets them and produces the appropriate command *C* for the robot. The command *C* is then sent to the robot via wired LAN. Based on the received command, the robot moves the end effector to the new position $p_{e}$. The end-effector position $p_{e}$ is visually observed by the operator. The feedback accuracy of the perceived position $p_{e,v}$ highly depends on the operator’s visual depth perception. Therefore, the operator has to have a clear view of the end effector and be relatively near (i.e., approximately at an arm’s length). Consequently, the operator has to be able to move freely around the robot.

### 2.2. Wearable IMU System

**Figure 2:**
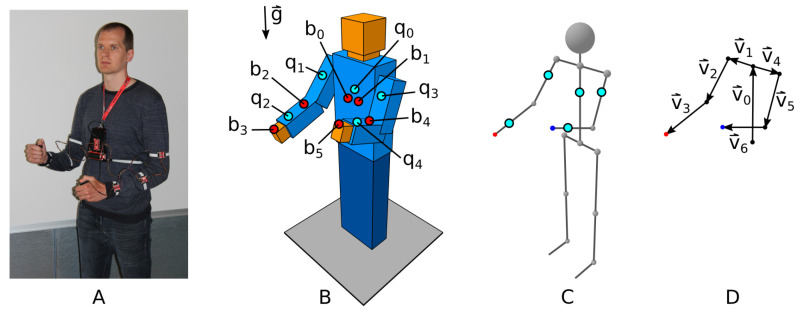
(**A**) The wearable IMU system, (**B**) the positions of IMUs and buttons, (**C**) the skeleton abstraction, and (**D**) the vector abstraction.

The wearable IMU system shown in Figure 2A is the first part of the Human–Machine Interface (HMI) of the developed system. The IMU system has a modular structure and consists of 5 IMUs, 6 buttons, a microcontroller with wireless capability and a battery power supply.

The IMUs used are Bosch’s BNO080 9DoF system-in-a-package (SiP) with CEVA’s Hillcrest Labs firmware for signal processing/fusion. Each IMU integrates a triaxial accelerometer, triaxial gyrometer, triaxial magnetometer, and a 32-bit ARM Cortex M0+ microcontroller. The IMU’s Gaming Rotation Vector mode is used to measure orientation in space $q_{i}$ in the form of a rotation quaternion, with a dynamic error of $2.5^{\circ }$, a static error of $1.5^{\circ }$, and a heading drift of $0.5^{\circ }$/min. The Gaming Rotation Vector mode does not use the magnetometer data and initializes the Z-axis based on gravity $\vec{g}$, while the X-axis is initialized freely.

The push-buttons are monitored by a dedicated microcontroller and controllably illuminated by a light-emitting diode (LED). Each button outputs data indicating its current state $b_{i}$. The buttons in the operator’s hands are used as momentary push-buttons. Other buttons are used as toggle buttons. The microcontroller is an Espressif’s ESP32 WROOM module with 240 MHz clock frequency of the Xtensa^®^ dual-core 32-bit LX6 microprocessor, 520 kB internal SRAM, and an integrated 802.11 BGN WiFi transceiver. The microcontroller is connected to the IMUs and buttons via i2c.

The wearable system elements on the operator’s torso (i.e., microcontroller, IMU, 2 buttons, and battery) are mounted using a small 3D-printed housing that is attached to the body with a neck strap and an elastic band around the back. The elements on the operator’s upper arms and forearms are also attached by elastic straps. The buttons in each hand are positioned on plastic holders attached to the operator’s wrists using string straps. The operator can release the button holders and use their hands at any time. The exact positions of the IMUs on the operator’s body are assumed to be unknown, and therefore, the mounting of the wearable system does not need to be precise. The described method for wearable system mounting is sufficient for prototype operation and satisfies the requirement for flexibility. However, it can be improved and made more robust and comfortable for prolonged use.

Data acquired with the wearable IMU system are used to describe the operator’s upper body using different abstractions, as shown in Figure 2. The positions of the IMUs on the operator’s body are depicted with light blue circles, and the buttons are indicated by red circles. The skeleton abstraction shown in Figure 2C is used in further figures to represent the operator’s position. The vector abstraction shown in Figure 2D is used by the control computer to calculate the approximate positions of the right and left hands.

**Figure 3:**
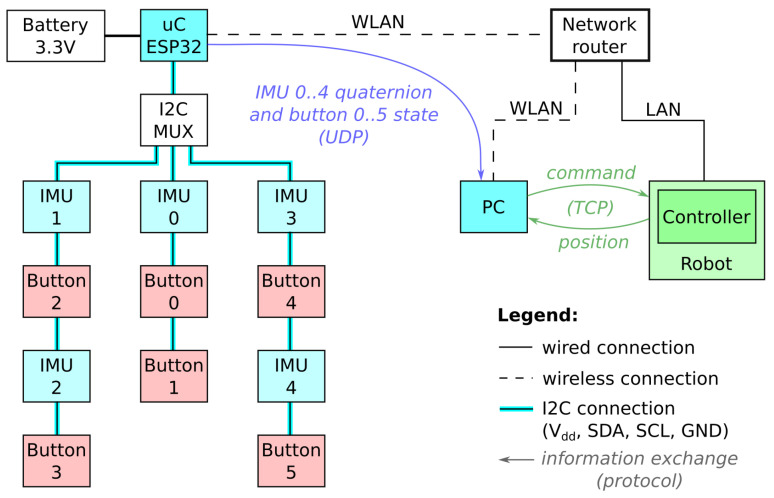
Connections and protocols.

The operator’s body orientations *q* and button states *b* are acquired by the microcontroller with a constant frequency of 30 Hz and sent to the computer for interpretation through User Datagram Protocol (UDP) messages, as shown in Figure 3 and Algorithm 1. With the use of UDP, the communication is faster but also less reliable. It is possible that messages never reach their destination, without the communicating devices becoming aware of them. When sending messages with high frequencies, it is also possible that messages arrive in an unpredictable order. For these reasons, the algorithm loop frequency is constant and much lower than the maximum possible measurement frequency (i.e., >100 Hz) achievable with the hardware used. Tests using a more reliable Transmission Control Protocol (TCP) revealed reoccurring latency spikes preventing timely message transmissions with constant frequency.
**Algorithm 1** Wearable IMU system: Rotation and button state acquisition.1:$f_{update}=30.0$ Hz2:initialize I2C mux, buttons, and IMUs (Game Rotation Vector output at 50 Hz)3:connect to WiFi4:**loop** with $f_{update}$5:      $b_{i}\leftarrow \mathrm{get}\_\mathrm{button}\_\mathrm{state}(i);i\in \left[0,6\right)$6:      $q_{i}\leftarrow \mathrm{get}\_\mathrm{IMU}\_\mathrm{rotation}\_\mathrm{quaternion}(i);i\in \left[0,5\right)$7:      send *q* and *b* as UDP message payload to address:port of control computer8:**end loop**

### 2.3. Control Computer

In the developed teleoperation system, a control computer is used to interpret the acquired data about the body position of the operator and the button state. The interpreted data are then transformed to a command for the collaborative robot and sent to it using an HTTP GET request that returns the robot’s position. The control computer runs Algorithm 2, which is designed to evaluate every received message from the wearable IMU system in a non-blocking manner.

The relative body position of the operator is calculated as changes Δq from base positions $q_{base}$ to measured positions *q*. However, because the IMUs’ coordinate systems are misaligned after initialization, a calibration procedure is necessary before measurements can be used to control the robot.

**Figure 4:**
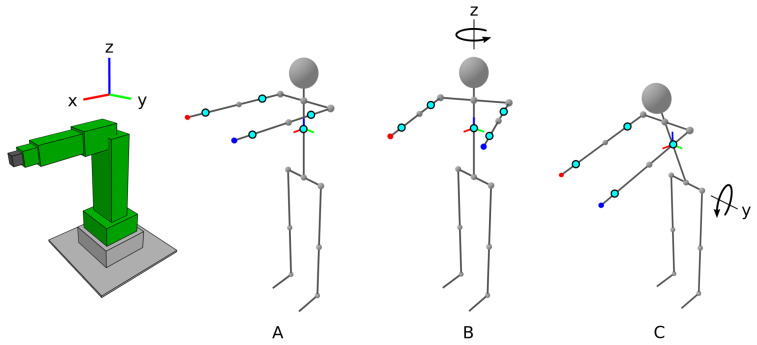
The calibration procedure. (**A**) The starting position of the operator, (**B**) the position for determining the *Z* axis and (**C**) the position for determining the *X* and *Y* axes.

The goal of the calibration procedure is to determine the correctional rotational quaternions $q_{corr_{Z}}$ and $q_{corr_{XY}}$, which are used to rotate the operator’s body orientation changes Δq to the corrected orientation $\Delta q_{corr}$ that would be measured if all IMUs’ local Cartesian coordinate systems were aligned with the robot’s global Cartesian coordinate system. The correction quaternions are based on three positions of the operator’s body during calibration, as shown in Figure 4.

The procedure starts after a button $b_{0}$ is pressed, with the operator standing upright with arms extended forwards and positioned in parallel, aligned with the X-axis of the robot’s global coordinate system (Figure 4A). With the first press of the right-hand button $b_{3}$, this position becomes the base position $q_{base}$. Then, the operator retains the body pose and only rotates around their vertical axis for a significant angle (e.g., $30^{\circ }$). The second position $q_{rot_{Z}}$ (Figure 4B) is confirmed by the second press of the right-hand button. Based on the second position, the vertical correction quaternions $q_{corr_{Z}}$ are determined using the following procedure, which produces a quaternion that aligns the Z-axis of the IMUs with the Z rotation axis (Figure 4B). For the difference quaternion $\Delta q=q_{rot_{Z}}\cdot q_{base}^{-1}=w+xi+yj+zk$, the cross and dot products of the vector corresponding to the Z-axis are calculated, as shown in Equation (1).

(1) $$\begin{array}{cc} a & =\frac{\left(x,y,z\right)}{\left|(x,y,z)\right|}\times \left(0,0,1\right)\\ \cos\theta_{xy} & =\frac{\left(x,y,z\right)}{\left|(x,y,z)\right|}\cdot \left(0,0,1\right). \end{array}$$

Then, the normalized quaternion that aligns the Z-axis of the IMUs is calculated as shown in Equation (2).

(2) $$q_{corr_{Z}}=\frac{\left[2\cdot \cos\theta_{xy},a_{x}i,a_{y}j,a_{z}k\right]}{\left|[2\cdot \cos\theta_{xy},a_{x}i,a_{y}j,a_{z}k]\right|}.$$

The correction quaternions are calculated for every IMU.

Next, the operator returns to the first position, where their arms are aligned with the robot’s X-axis. The operator again retains the body pose and only rotates their upper body part around the horizontal axis with their hips aligned with the robots’s Y-axis (i.e., the operator bows down) for a significant angle (e.g., $30^{\circ }$). The third position, $q_{rot_{XY}}$ (Figure 4C), is confirmed by the third press of the right-hand button. Based on the third position, the horizontal correction quaternions $q_{corr_{XY}}$ are determined using the following procedure, which determines the quaternion that aligns the axis of upper body rotation with the Y-axis. The procedure looks for a quaternion that represents a pure rotation around the Z-axis for a half-angle $\theta_{Z}$, shown in Equation (3).

(3) $$q_{corr_{XY}}=\cos\theta_{z}+0i+0j+\sin\theta_{z}k.$$

If the difference between the IMU orientations in the first and third positions is denoted as $\Delta q=q_{rot_{XY}}\cdot q_{base}^{-1}=w+xi+yj+zk$, the half-angle required for aligning the axis can be calculated as shown in Equation (4).

(4) $$\theta_{z}=\arctan\left(y,x\right)/2.$$

Of course, the alignment of the operator with the robot is not ideal; however, the procedure is precise and reliable enough for intended use. It is important that the calibration can be performed quickly and without additional equipment. The calibration procedure can be restarted, if needed, by pressing the button $b_{0}$.

The corrected orientation quaternion changes $\Delta q_{corr}$ are used to set the model vectors *v* of the operator’s upper body in the corresponding position $v_{rotated}$, as shown in Equation (5), where $\Delta q=q\cdot q_{base}^{-1}$.

(5) $$\begin{array}{cc} \Delta q_{corr} & =q_{corr_{XY}}\cdot \left(q_{corr_{Z}}\cdot \Delta q\cdot q_{corr_{Z}}^{-1}\right)\cdot q_{corr_{XY}}^{-1}\\ v_{rotated} & =\Delta q_{corr}\cdot v\cdot \Delta q_{corr}^{-1}. \end{array}$$

The vector model is a relatively crude approximation of a human body, but it is accurate enough if the vector lengths are adjusted for individual operators. Based on the sum of the appropriate rotated vectors $v_{rotated}$, hand locations $p_{local}$ relative to the operator’s local coordinate system are determined. Because the robot is controlled by body movement, the change Δp between current $p_{local}$ and previous $p_{base}$ hand locations are calculated and stored. Base hand locations $p_{base}$ are updated with new locations $p_{local}$ for every received message.

After every n-th (e.g., 3rd) reported position of the operator, the state of hand buttons $b_{3}$ and $b_{5}$ is checked for robot control intention, and the control button $b_{1}$ is checked for robot control permission. A moving average of m (e.g., 3) stored hand location changes $\Delta p_{avg}$ is then appropriately amplified with a linear factor *a* to produce robot movement control inputs CX. For the control of rotation with the operator’s left hand, the largest movement is isolated, because it was experimentally determined that rotating the robot’s end effector in only one axis at a time is more intuitive to the operator.

A very important condition for flexible robot teleportation is the synchronization of the operator’s local coordinate system and the robot’s user frame (UF). The result of synchronization is the aligned movement direction of the operator and the robot. For synchronization, only rotations of the user frame and tool frame are taken into account.

Another important consideration for robot teleportation is ensuring that the robot movement is smooth, which can be achieved by maintaining the robot’s movement speed in exchange for positional precision. The proportion of speed that is maintained at a target position is defined by a speed conservation factor cnt, which is proportional to the command input and adds virtual inertia to the robot’s movement that increases the movement smoothness. In our case, it is set linearly proportional to the absolute maximum change in the desired end-effector position. This results in a smooth, fast movement and a precise, slow movement.

At the end, an HTTP GET request is compiled and sent to the robot based on the desired gripper state determined by $b_{2}$, speed conservation factor cnt, movement control inputs CX, and user frame UF. The robot responds with its current position in joint space and in the user-frame-based Cartesian space, which are saved for further analysis.
**Algorithm 2** Control computer: Transformation of rotations to commands.1:initialize variables2:**loop**3:      receive UDP_message from Wearable IMU system4:      **for** each button **do**5:    save previous button state $b_{base}=b$6:    get new button state *b* from UDP_message7:      **end for**8:      **for** each IMU **do**9:    get IMU quaternion *q* from UDP_message10:     calculate change Δq between *q* and $q_{base}$11:     corrected change $\Delta q_{corr}$ based on $q_{corr\_Z}$ and $q_{corr\_XY}$12:    **end for**13:    calibration procedure interface (get $q_{base}$, $q_{corr\_Z}$ (Equation (2)), and $q_{corr\_XY}$ (Equation (3)) based on $\Delta q_{corr}$)14:    **for** each *v* **do**15:     rotated model vector $v_{rotated}$ based on corresponding *v* and $\Delta q_{corr}$16:    **end for**17:    hand positions *p* based on sums of $v_{rotated}$ subsets18:    send data for 3D visualization of human operator19:    local hand positions $p_{local}$ are equal to rotated *p* with inverse $\Delta q_{corr}$ for torso20:    hand position change Δp between $p_{local}$ and $p_{base}$21:    update Δp history22:    $p_{base}$ = $p_{local}$23:    **if** $b_{3}=0\vee b_{3,base}=0$ **then**24:    reset Δp history for right hand25:    **end if**26:    **if** $b_{5}=0\vee b_{5,base}=0$ **then**27:    reset Δp history for left hand28:    **end if**29:    i=i+130:    **if** i=n **then**31:      i=032:      **if** $b_{1}=1\wedge \left(b_{3}=1\vee b_{5}=1\right)$ **then**33:      moving average hand location change $\Delta p_{avg}$ for last *m* points in time34:      control inputs CX based on $\Delta p_{avg}\cdot a$35:      isolate max(abs(CX)) only for rotation36:      determine cnt based on max(abs(CX))37:      user frame UF based on $\Delta q_{corr}$ for operator’s torso38:      compile GET_request based on gripper_button, cnt, CX and UF39:      send GET_request to robot controller40:      **end if**41:    **end if**42:**end loop**

### 2.4. Collaborative Robot

The collaborative robot used in the developed system is a Fanuc CR-7iA/L robot with an R-30iB Mate Plus controller. The robot has a typical 6-axis industrial robot configuration, a maximum payload of 7 kg, and a reach of 911 mm. The robot can be programmed with a Teach Pendant (TP) and KAREL programming language.

The robot receives the HTTP GET request through the developed KAREL program and updates specified registers with new values, as shown in Algorithm 3. The expected update frequency is 10 Hz. The updated registers are used by a separate TP program to move the robot.

The register update sets the gripper state, speed maintenance factor cnt, and position-related registers. The user frame and tool frame are set to match the orientation of the operator. Based on the current position of the end effector in Cartesian space and the requested change, a new position is calculated. The ability of the robot to reach the new position is checked to avoid errors during movement attempts. At the end of the program, the robot’s current position is sent as a reply to the control program.
**Algorithm 3** Collaborative robot: Register update based on received request.1:  receive GET_request from Control computer2:  get cnt, gripper_state, $\Delta position_{X}$, user_frame from GET_request3:  $cnt_{R}=cnt$4:  $gripper\_state_{R}=gripper\_state$5:  $user\_frame_{PR}=user\_frame$6:  $tool\_frame_{PR}=\left(0,0,tool\_length,user\_frame.w,-user\_frame.p,-user\_frame.r\right)$7:  get $position_{X}$ in Cartesian space8:  get $position_{J}$ in Joint space9:  $new\_position_{X}=position_{X}+\Delta position_{X}$10:**if**$new\_position_{X}$ is not reachable **then**11:       $new\_position_{X}=position_{X}$12:**end if**13:$position_{PR}=new\_position_{X}$14:respond to GET_request with ($position_{X}$ and $position_{J}$) in JSON format

To move the robot, a TP program, described in Algorithm 4, is used. At the beginning of the program, the user frame and tool frame are defined. The robot’s maximum movement speed is defined for linear and rotational movements with a combination of $speed_{R}$ and $override_{R}$ parameters. The desired position $position_{PR}$ is initialized as the current position of the robot’s end effector. Then, in a continuous loop, the gripper is closed or opened, and the end effector is moved to the desired position. The movements of the individual robot joints are determined autonomously by the robot according to its default inverse kinematics algorithm, which takes into account the speed conservation factor. It is important to note that the movement needs to be fully completed before the loop continues. The time needed to complete the motion depends on the change in position, start speed, end speed, and acceleration. Ideally, the motion time should be equal to the control period (i.e., 100 ms). In parallel, the collaborative robot also monitors the exerted load onto its structure and immediately aborts the robot motion if the load exceeds permitted values.
**Algorithm 4** Collaborative robot: Register-based motion control.1:set user_frame_number2:set tool_frame_number3:$speed_{R}=const.$4:$override_{R}=const.$5:$position_{PR}$ is equal to current position in Cartesian space6:**loop**7:      set output $gripper\_state_{OR}=gripper\_state_{R}$8:      make linear move to $position_{PR}$ with $speed_{R}$ and $cnt_{R}$9:**end loop**

Both programs that run on the robot are Fanuc-robot specific; however, it should be possible to implement the described functionality on any comparable industrial robot.

### 2.5. Flexible Teleoperation

**Figure 5:**
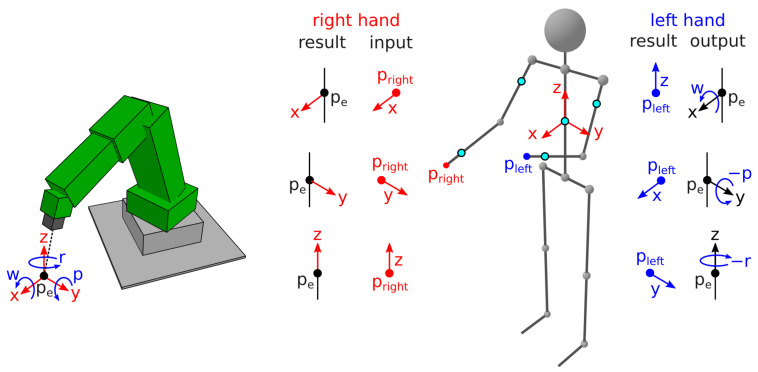
The robot controls using the developed teleoperation system.

The developed teleoperation system allows the operator to move the robot’s end-effector position with the change in hand locations, as shown in Figure 5. Translatory movement of point $p_{e}$ is entirely controlled by the operator’s right hand, while the control over rotational movement is performed by the left. However, functions dedicated to the hands can easily be switched if that is preferred by the operator.

The robot only moves if its control is enabled by the control button $b_{1}$. The translatory motion of the end effector $p_{e}$ only occurs if the right-hand button $b_{3}$ is pressed and the location of the right hand $p_{right}$ changes relative to the operator’s torso. The rotational motion only occurs if the left-hand button $b_{5}$ is pressed and the location of the left hand $p_{left}$ changes relative to the operator’s torso.

The relation between the location of the right hand $p_{right}$ and that of the end effector $p_{e}$ is trivial. The movement direction of the end effector is always aligned with the movement direction of the right hand. The translatory movement magnitude of the end effector depends on the amplified translatory movement magnitude of the right hand.

The relation between the location of the left hand $p_{left}$ and end-effector rotation is more complex but still intuitive. The orientation of the user and the tool frame of the robot is equal to the orientation of the operator’s torso or its local coordinate system. To rotate the end effector around the X-axis or to change W rotation, the left hand is moved in the Z-axis (e.g., up or down). To rotate the end effector around the Y-axis or to change P rotation, the left hand is moved in the X-axis (e.g., forward or backward). To rotate the end effector around the Z-axis or to change R rotation, the left hand is moved in the Y-axis (e.g., left or right). The directions of P and R rotations are reversed because the arm motions correspond better to the motion of the end effector.

The described relations are true regardless of the operator’s position around the robot. This allows the operator to simplify their arm motions by moving to a more suitable control location or orientation around the robot. Because the operator’s control feedback loop is visual, the operator can also change their location to acquire a better view of the end effector and improve the precision of the control.

## 3. Results

The main result of this research is a working wearable IMU system for the flexible teleoperation of a collaborative robot. To demonstrate and evaluate the teleoperation flexibility of the developed system, an example of its use is presented.

### 3.1. Evaluation Setup

**Figure 6:**
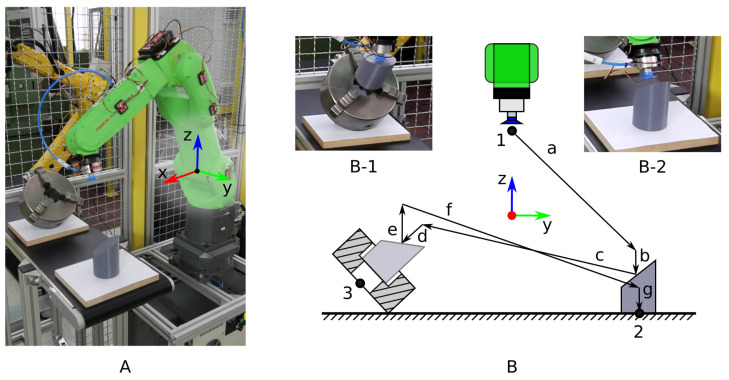
(**A**) The evaluation setup with a collaborative robot, workpiece, and fixture. (**B**) The view of the main movement plane. (**B-1**) The insertion of the workpiece in the fixture. (**B-2**) The approach to the workpiece.

The example used in this study is a case of workpiece manipulation by an operator with the use of a collaborative robot. The operator has to move a workpiece to a fixture and back to its original position, as shown in Figure 6. The workpiece is a truncated cylinder, and the fixture is a lathe chuck. A pneumatic suction cup is used to pick up the workpiece.

The experiment was designed to require combined movement in multiple axes of Cartesian space to approach the desired path a–g and move back in the reverse order g–a. The start and end position of the robot’s end effector is position 1, where the end effector is positioned above and closer to the robot’s origin than the workpiece and the fixture. The workpiece, a truncated cylinder, weighs 400 g, has a diameter of 80 mm, a maximum height of 120 mm, and a top face with a slope angle of $55^{\circ }$. The workpiece starts at position 2, with the top face oriented towards the fixture at position 3. The opening of the fixture is pointed upwards at a $50^{\circ }$ angle and towards the initial workpiece position. Its jaws have a height of 30 mm and are opened to accept cylindrical objects with a slightly larger diameter than that of the workpiece (i.e., 85 mm).

The evaluation task is divided into several subtasks. The robot’s end effector starts at position 1 and is then moved to the workpiece at position 2, where the workpiece is picked up with the suction cup. The workpiece is then moved to the fixture at position 3. The workpiece is inserted in the fixture and left there while the end effector is moved to position 2 to touch the surface for a few seconds. Afterwards, the end effector is moved back to position 3, and the workpiece is extracted from the fixture and returned to its original position. The end effector is finally moved to position 1, where the evaluation task is concluded. The evaluation task consists of various different robot motions in a sequence of sufficient complexity and duration to show the properties of the developed system. The use of the vacuum cup to hold the workpiece also adds a level of difficultly, because the workpiece weight can cause the cup to deform, resulting in a sag that depends on the angle at which the workpiece is held.

### 3.2. Standard Robot Control

For the purpose of comparison, two reference robot motions during the evaluation task execution were observed. The two motions can be achieved with a standard robot interface and control equipment.

**Figure 7:**
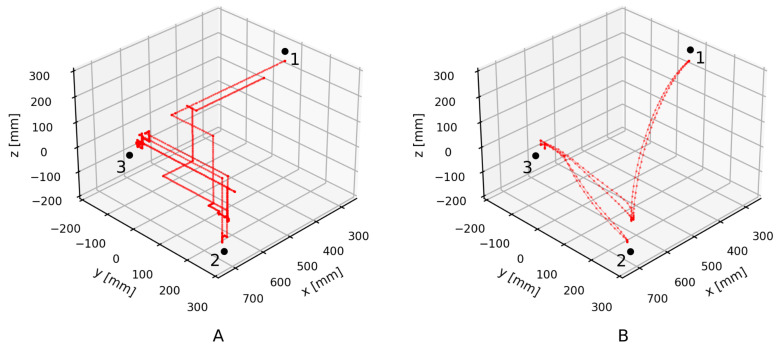
Translation of the robot’s end effector during the evaluation task (**A**) when the operator is manually controlling the robot with a teach pendant and (**B**) when the robot is in automatic mode controlled by a program.

Firstly, the robot is controlled manually by an operator using a teach pendant. The teach pendant has a size of 340 × 200 × 70 mm and weighs 1390 g. The operator holds the teach pendant with one hand, which is also pressing the dead man’s switch, and presses the command keys with the other hand. The robot moves according to keypad presses and a set movement speed. The operator controls the robot in Cartesian space, one axis at a time. The robot path shown in Figure 7A is consequently constructed of straight perpendicular segments that have stationary points between them. The robot only moves when a control key is pressed and therefore halts between key presses. Smooth movement in multiple spatial dimensions can only be roughly approximated using short alternating movements in different directions. This type of motion is used to insert the workpiece in the fixture at point 3. However, precise movement in more than one dimension is hard to achieve.

Secondly, a control program is created for the robot by the operator using the teach pendant. Execution of the program produces smooth movement, as shown in Figure 7B. Motion speed is maintained wherever possible. The robot motion is precise and efficient. However, to create the program, the operator has to manually move the robot to key positions on the path and save them in registers. Additionally, the program is only useful if the workpiece and the fixture have repeatable starting positions. Therefore, program creation for non-repeatable situations, which are the focus of this research, is highly impractical.

Finally, after observing the two robot motion examples, the desired robot motion can be described as a compromise between them. The robot motion should be relatively precise and smooth but also adapted to specific situations with real-time control.

### 3.3. Flexible Robot Control

**Figure 8:**
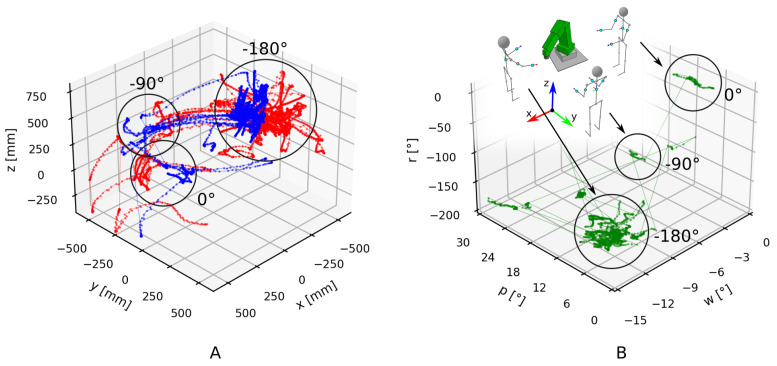
(**A**) Translation of the operator’s left (blue) and right (red) hands, and (**B**) rotation of the operator’s body while performing the evaluation task with the developed IMU system.

**Figure 9:**
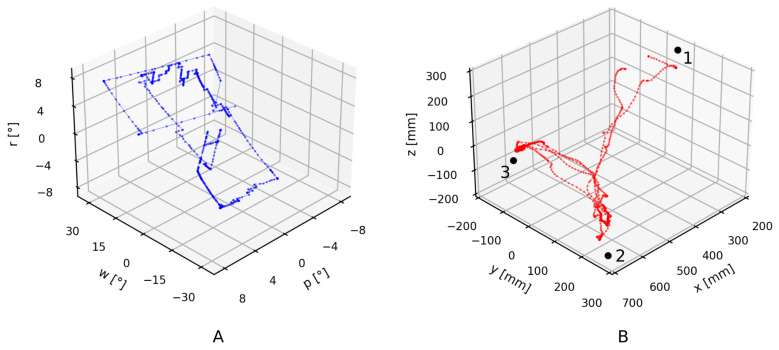
(**A**) Rotation of the robot’s end effector, and (**B**) its translation while the evaluation task was performed with the developed IMU system.

The developed wearable IMU system is designed to provide the operator with more flexibility while controlling the collaborative robot in comparison with standard robot control. The increase in flexibility comes from the intuitive connection between the operator’s and robot’s motion. The connection is based on the measurement of the operator’s upper body rotations with IMUs. Information about the operator’s hand translations in Figure 8A and the rotation of the operator’s torso in Figure 8B is used to control the robot, resulting in the movement shown in Figure 9. Translations and rotation are shown in their entirety for the whole performance of the evaluation task. However, only when the buttons in the left or right hand are pressed is the information transformed into commands for the robot movement.

Translation of the operator’s hands, shown in Figure 8A, is relative to the operator’s torso. When the operator rotates, the hand positions translate accordingly. Translation of the operator without rotation has no effect on the perceived hand positions. Translation of the left hand, which controls the robot’s end-effector rotation, is shown in blue, and translation of the right hand, which controls the end-effector translation, is shown in red.

The rotation of the operator’s torso, shown in Figure 8B, controls the rotation of the robot’s coordinate system, that is, the user frame. At the beginning of the evaluation task, the operator stood beside the robot facing in the direction of the X-axis. Their torso rotation around the Z-axis, that is, r, was $0^{\circ }$. Later, the operator moved and rotated around the robot to view it from the side, $-90^{\circ }$ to the starting orientation, and then moved for the second time to view the robot from the front, $-180^{\circ }$ to the starting orientation. Rotations other than around the vertical axis (w and p) were considerably smaller and mostly a consequence of changes in the operator’s body posture. However, the operator did not notice their influence during teleoperation.

The robot’s end-effector rotation during the evaluation task, shown in Figure 9A, changed into distinct linear segments. Isolation of rotation commands relative to the user frame was intentionally implemented to better clarify the rotations intended by the operator. The rotations shown in Figure 9A are linear but also occur in multiple rotational axes at the same time. This is because the fixed coordinate system (i.e., global coordinate system) shown in the figures differs from the variable coordinate system of the robot and the operator (i.e., user frame). In addition, the majority of rotation occurred in a position $-180^{\circ }$ to the starting orientation of the operator, because it offered the best view of the workpiece and the fixture. Nevertheless, it can be observed that the dominant rotation of the end effector was around the X-axis (i.e., rotation w), in accordance with expectations for the evaluation task.

The robot’s end-effector translation, shown in Figure 9B, was much smoother in comparison to when the operator used the teach pendant to control the robot (Figure 7A); however, there was still some waviness in longer translation segments. This occurred because the operator’s hand translation was scaled down, and the operator sometimes needed to combine multiple hand translations into one longer end-effector translation. Consequently, shorter translations were more precise. The mode of precision can be changed by pressing a dedicated button, but that also creates a pause in the motion, which is a source of the waviness. Simultaneously achieving full range and full precision of the operator’s hand motion would be difficult, because the time delay in the control loop, consisting of the human and machine parts, during precise positioning could result in end-effector oscillation.

**Figure 10:**
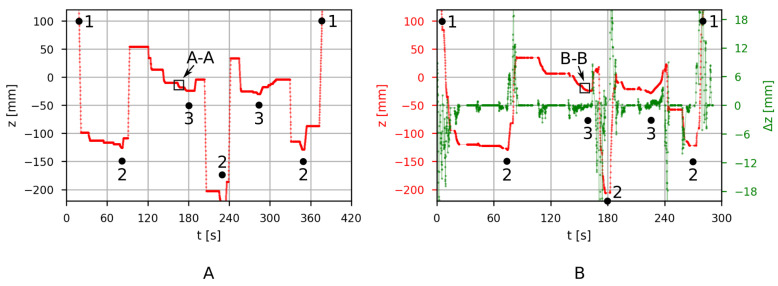
Translation (red) of the robot’s end effector in vertical direction (i.e., Z-axis) (**A**) when the operator is manually controlling the robot with a teach pendant and (**B**) when the operator is manually controlling the robot with the developed IMU system. Input (green) for the robot from the developed IMU system is a change in position Δz.

Translation of the end effector in the Z-axis during the evaluation task is shown in Figure 10A when the robot is controlled with a teach pendant and in Figure 10B when controlled with the developed wearable system. Because the same task is performed in both cases, the overall shape of the two translation graphs is similar. With the developed system, the operator was able to complete the task approximately 20% faster than the experienced operator using a teach pendant. Further experiments are required to evaluate the improvements in operator efficiency, however, this is not the main objective of the developed system. From the initial experiments, it is clear that the benefits grow as the complexity of the task increases. The main reason for this is that simultaneous movement in multiple spatial axes is much easier and more intuitive with the developed system compared to the teach pendant. This is further illustrated near position 3, when the workpiece is inserted into the fixture.

**Figure 11:**
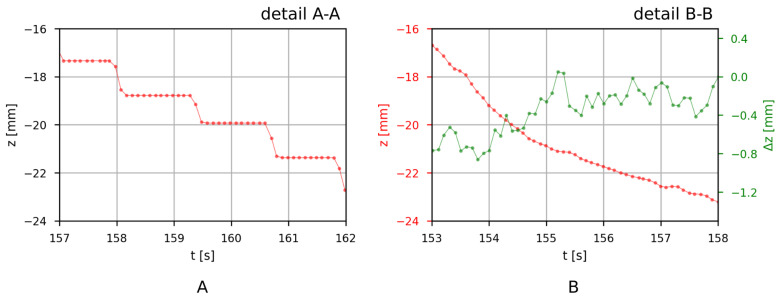
Translation (red) of the robot’s end effector in the vertical direction (i.e., Z-axis) (**A**) for detail A-A in Figure 10 when the operator is manually controlling the robot with a teach pendant and (**B**) for detail B-B in Figure 10 when the operator is manually controlling the robot with the developed IMU system. Input (green) for the robot from the developed IMU system is a change in position Δz.

The insertion subtask detail A-A for the teach pendant is shown in Figure 11A, and detail B-B for the developed wearable system is shown in Figure 11B. Translation made with the teach pendant at this time interval is a sequence of alternating translations in the Z- and Y-axes in order to move the workpiece at an angle relative to the work surface. The same subtask was also achieved with the wearable system, which supports simultaneous translation in all axes. The resulting translation in comparison with the teach pendant case is much smoother and consequently has higher precision. Figure 11B also shows the command input for the robot in green. The input tells the robot how much it should move in the Z-axis. However, the resulting translation also depends on the speed conservation factor (variable), robot acceleration (constant), and desired final speed (constant).

The flexibility during teleoperation is based on the ability of the operator to freely move around the robot without the need to think about the orientation of the robot’s coordinate system. Furthermore, the operator can move to a position that allows for the simplification of the control hand motions. For example, if the workpiece needs to be moved precisely along a line at an angle relative to the operator’s current orientation, the operator only needs to align with the line of the desired motion. Consequently, 2D hand motion is simplified to 1D motion, which achieves the same robot movement. This is also true for rotations. If rotation is desired around a specific axis, the operator can align with the axis and perform a pure 1D rotation.

## 4. Discussion

### 4.1. Safety and Real-Time Considerations

Safety in the work system is first ensured by using a collaborative robot that will stop if the detected load exceeds the allowed values. The second safety feature is the fault-tolerant design of the wearable system. The robot moves only if it receives a valid command for a relatively short movement to a location that the robot can reach. If communication between the elements is interrupted, then the robot will fulfill the last command and halt until it receives a new valid command. Valid position change is limited, preventing fast undesirable robot motion. Therefore, if an IMU measurement is incorrect/erratic for some reason, the system will show an unexpected/strange disparity between the desired motion and the observed motion, allowing the operator to safely detect and abort the control by releasing the control button.

Furthermore, the system design takes into account the problems associated with real-time control of robotic systems, such as communication delays and packet loss. In the experimental setup, the response time between the microcontroller and the PC was 2.93 ms (st.dev.1.04 ms), on average. In the presented experiment, the packet loss via UDP was 4/9579=0.04%. Additionally, network issues are mitigated by the redundancies in the processing pipeline. Sensor data is acquired at 50 Hz, sent to the PC at 30 Hz, and then, in the current implementation, three messages are considered before sending the command to the industrial robot at 10 Hz. This provides higher system reliability, which is needed in industrial applications. In terms of processing power, the limitation is the microcontroller. Nevertheless, the microcontroller loop uses only 33.0% of the time for sensor data acquisition and 9.5% for sending the data over WiFi, while the rest (57.5%) is spent idle.

### 4.2. Limitations and Future Work

The wearable system is designed for flexible teleoperation of an industrial robot. However, the characterization of flexibility is somewhat problematic. The results clearly show that the developed system allows for a more flexible control of the robot than if the operator were to use a standard robot controller, that is, a teach pendant. However, it is difficult to know the exact extent of the difference. A robot teleoperation flexibility measurement, which, in addition to the robot’s motion, takes into account the state of the operator (e.g., stress level based on heart rate or brain activity measurements with electroencephalography (EEG)), would have to be implemented to support further improvement of the system.

The most beneficial improvement would be an implementation of automatic motion scaling to achieve larger motion ranges and higher precision, without the possibility of feedback-induced oscillation. The precision depends on the scaling factor (gain), which scales the motion of the robot’s end-effector in relation to the motion of the hands. A predictive algorithm to automatically adjust the gain would further improve the flexibility of the system. The wearable system could also be integrated into the operator’s work clothing, simplifying the IMU mounting. The torso IMU should then be repositioned to avoid slight interference with the orientation of the user frame from chest movements caused by breathing. An additional haptic feedback loop could be added to allow the operator to control the robot’s end-effector contact force and improve the interaction with objects in the workspace.

## 5. Conclusions

The developed wireless wearable system supports the flexible teleoperation of collaborative robots. The system captures the operator’s hand movements through a system of IMUs and translates them into commands for the robot’s controller. The presented approach solves several problems: (1) a calibration procedure is devised that aligns the coordinate systems of IMUs to the coordinate frame of the robot; (2) a method for transforming the hand movements into commands for the robot is proposed; and (3) communication and safety issues are addressed in the context of an industrial collaborative robot. The flexibility of teleoperation is then improved by adjusting the operator’s frame of operation depending on his orientation, in real time. This in turn enables intuitive interactions where the operator, for example, moves their hand in a straight line, which is then translated into a straight motion in all spatial axes of the robot’s frame. The fact that the operator can move freely around the robot is highly beneficial and contributes to the usability of the wearable system as a human–machine interface. The experimental results confirm the feasibility of the approach and the usability of the system.

## Data Availability

Not applicable.
